# Nuclear Segmentation in Histopathological Images Using Two-Stage Stacked U-Nets With Attention Mechanism

**DOI:** 10.3389/fbioe.2020.573866

**Published:** 2020-10-26

**Authors:** Yan Kong, Georgi Z. Genchev, Xiaolei Wang, Hongyu Zhao, Hui Lu

**Affiliations:** ^1^SJTU-Yale Joint Center for Biostatistics and Data Science, Department of Bioinformatics and Biostatistics, School of Life Sciences and Biotechnology, Shanghai Jiao Tong University, Shanghai, China; ^2^Center for Biomedical Informatics, Shanghai Engineering Research Center for Big Data in Pediatric Precision Medicine, Shanghai Children’s Hospital, Shanghai, China; ^3^Bulgarian Institute for Genomics and Precision Medicine, Sofia, Bulgaria; ^4^Department of Biostatistics, Yale University, New Haven, CT, United States

**Keywords:** nuclei segmentation, histopathological image, Stacked U-Nets, attention generation mechanism, deep learning

## Abstract

Nuclei segmentation is a fundamental but challenging task in histopathological image analysis. One of the main problems is the existence of overlapping regions which increases the difficulty of independent nuclei separation. In this study, to solve the segmentation of nuclei and overlapping regions, we introduce a nuclei segmentation method based on two-stage learning framework consisting of two connected Stacked U-Nets (SUNets). The proposed SUNets consists of four parallel backbone nets, which are merged by the attention generation model. In the first stage, a Stacked U-Net is utilized to predict pixel-wise segmentation of nuclei. The output binary map together with RGB values of the original images are concatenated as the input of the second stage of SUNets. Due to the sizable imbalance of overlapping and background regions, the first network is trained with cross-entropy loss, while the second network is trained with focal loss. We applied the method on two publicly available datasets and achieved state-of-the-art performance for nuclei segmentation–mean Aggregated Jaccard Index (AJI) results were 0.5965 and 0.6210, and F1 scores were 0.8247 and 0.8060, respectively; our method also segmented the overlapping regions between nuclei, with average AJI = 0.3254. The proposed two-stage learning framework outperforms many current segmentation methods, and the consistent good segmentation performance on images from different organs indicates the generalized adaptability of our approach.

## Introduction

Morphological changes in the cell nucleus are considered an important signal in many diseases (Gurcan et al., 2009) and can provide clinically meaningful information during diagnosis, especially for cancers (Chow et al., 2015). The conventional method involves manual inspection and analyses performed by pathologists to make diagnostic assessments based on certain morphology features of the nucleus. However, this manual assessment is a tedious and time-consuming task that can be beset by shortcomings such as poor sensitivity, specificity, and low reproducibility. This fact underscores the urgent need to develop and refine rapid and automated histology image analysis methods; nuclear segmentation is often the most important and fundamental one (Fuchs and Buhmann, 2011).

Deep neural networks, especially deep convolutional neural networks (CNNs), have been the dominant techniques for visual analysis and have recently achieved great success for biological object detection and segmentation in medical images (Aoki et al., 2020; Wen et al., 2020; Xiang et al., 2020; Yao et al., 2020; Zhang et al., 2020). U-Net (Ronneberger et al., 2015) is a classical architecture based on fully convolutional network (FCN) (Long et al., 2015), which has been widely used and has obtained promising performance when applied to the task of image segmentation (Litjens et al., 2017; Kong et al., 2020). Furthermore, many studies have improved the original U-Net, such as Res-Unet (Xiao et al., 2018) or dense-Unet (Zhu et al., 2019). Among many improved networks, multi-scale and stacked networks have attracted intensive studies. For example, Wu et al. (2018) utilized the multi-scale network followed networks (MS-NFN) model to segment blood vessels in retinal images, while Sevastopolsky et al. (2018) proposed a special cascade network which stacked two kinds of blocks, U-Net or Res-UNet, for optical disc and cup segmentation. On the other hand, Stacked U-Nets (SUNets) (Shah et al., 2018) can be considered as further improvement as they iteratively combine features from different image scales while maintaining resolution. Leveraging the feature computation power of U-Nets in a deeper network architecture, SUNets are capable of handling images with increased complexity.

Due to the complexity of nuclei shape, imperfect slide preparation or staining, overlapping nuclei, and scanning artifacts, automatic nuclei instance segmentation is still a computationally challenging task. Compared to manual nuclei segmentation, however, automated segmentation methods based on cutting-edge deep learning technology have the potential to foster improvement.

Inspired by the attention mechanism idea (Vaswani et al., 2017) and the aforementioned segmentation approaches, we developed a two-stage learning framework based on two SUNets to solve the challenges in nuclei segmentation in histopathological images. We converted the nuclei segmentation task into a two-stage task; both stages were composed of a SUNets with the same architecture. The outputs of our SUNets were then post-processed through a watershed algorithm (Roerdink and Meijster, 2000) to achieve the instance-level segmentation. We also compared our method with current existing popular algorithms. When applied to a publicly available multi-organ dataset, our method achieved improved segmentation accuracy results and solved the segmentation challenge of overlapped nuclei regions with high fidelity. In addition, we applied our method on another publicly available dataset and obtained reliable segmentation results as well. Details of our method are described in section “Methodology”, comparisons on two independent image sets are elaborated in section “Results and Discussions”, and finally, conclusion is presented in section “Conclusion.”

## Methodology

**FIGURE 1:**
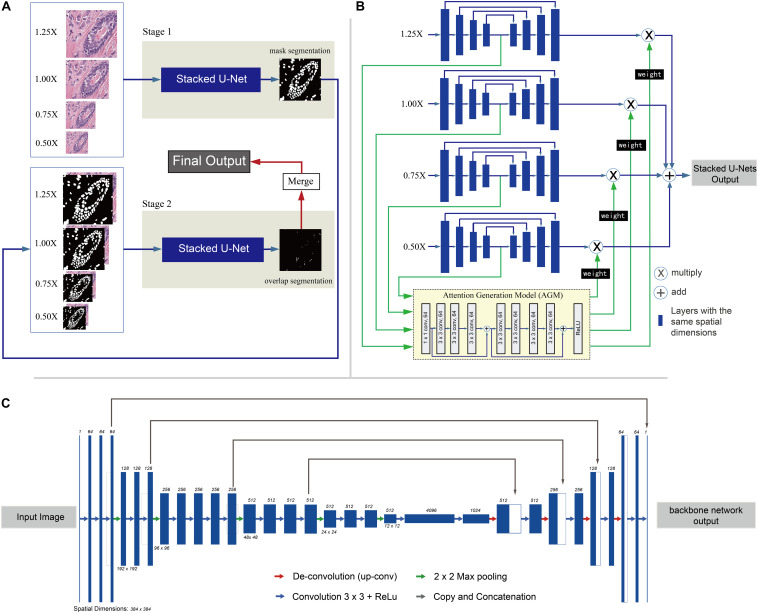
Overview of the two-stage learning method. **(A)** Overall flow chart of the two-stage method. Input: First, images are split into small patches of 384 × 384-pixel size, resized into four different scales (1.25×, 1.0×, 0.75×, and 0.50×). Stage 1: In stage 1, the patches are fed into the first set of Stacked U-Nets for the first round of nuclei segmentation. The Stacked U-Nets consist of four parallel backbone nets that have different sized images as input. At the end of stage 1, the mask segmentation of nuclei regions is generated with pixel gray value of 0 (not nuclei regions) and 1 (nuclei region). In addition, the nuclei instance segmentation is also predicted by the watershed algorithm. Stage 2: The stage 2 input contains not only the original RGB image patches but also the binary masks segmentation of nuclei regions predicted by stage 1 as the fourth set of values. At the end of stage 2, the segmentation result of overlapped regions is generated with pixel gray value of 0 (not overlapped regions) and 1 (overlapped region). Merge: In the merge step, the first round of nuclei instance segmentations results from stage 1 are updated by merging the corresponding overlapped objects, which have at least 10 pixels overlapped with the contour objects derived from stage 2. Output: The final output of the flow is nuclei instance segmentation result which includes separate nuclei of the overlapping regions if they have. **(B)** Architecture of the Stacked U-Nets. Blue rectangles stand for the multiple layers in the backbone net with the same spatial dimensions. The Attention Generation Model (AGM) is used to weight and sum the predictions of the four scaled backbone nets and generate the final segmentation. The output of the Attention Generation Model (AGM) is a weight matrix which weights for each backbone net that have different scaled images as input. Each backbone net returns a segmentation result weight matrix generated by the AGM which is used to multiply (X circle in panel **C**) the result of each segmentation result and sum them together (+ circle in panel **C**) to get the final result. **(C)** Detailed architecture of the backbone net used in the Stacked U-Nets. Each dark blue box corresponds to a multi-channel feature map. The number of channels is denoted on the top of the box. The spatial dimensions are provided under some of the boxes (boxes with the same height have the same spatial dimension). White boxes represent copied feature maps from layers where the gray arrows originate. The arrows with different colors denote the different operations–red for de-convolution, green for max-pooling, blue for regular convolution, and gray for copy and concatenation.

In this work, a two-stage framework is proposed to automate segmentation of nuclei regions and regions of overlapping nuclei. The flow of the two-stage framework is shown in Figure 1A. Two SUNets (Sevastopolsky et al., 2018; Figure 1B) with the same architecture are utilized in both stages. The first stage aims to segment nuclei regions, and the second stage is designed to segment regions of overlapping nuclei. Nucleus instance segmentation results from the first stage are updated by adding overlapped regions derived from the second stage. In this section, we present our two-stage method in detail.

### Segmentation of Nucleic Regions

Stage 1 in our nuclei segmentation framework predicts the region of each nucleus using CNNs, specifically SUNets. The SUNets consist of four parallel backbone nets. Four images derived from the original histopathology image by scaling at 1.25×, 1.0×, 0.75×, and 0.50× serve as inputs to Stage 1. Four semantic feature maps of differently sized backbone nets are extracted and fed into an attention generation module, which contains eight CNN layers belonging to the first two blocks of ResNet34 (Gulshan et al., 2016) together with one ReLU (rectified linear unit) layer. Finally, a weight matrix for each scaled backbone net is returned and used to weight and sum the predictions of the four scaled backbone nets and generate the final result. The architecture of the backbone net of our SUNets is illustrated in Figure 1C. The backbone nets which extract semantic information from the input image is based on a modified deep network–VGG16 (Wang et al., 2015). The down-sampling part of the network (the first 21 layers from the left side in Figure 1C) contains a series of convolutional layers with ReLU activation function (Nair and Hinton, 2010). The last down-sampling layer represents the semantic features of the input image. At the end of Stage 1, the mask segmentation of nuclei regions is generated. Image features are transformed into same-sized mask segmentation result with pixel gray value of 0 (background region) and 1 (nuclei region). Then the watershed algorithm is utilized to get the first round of nucleus instance segmentation result.

### Segmentation of Overlapped Nuclei Regions

Stage 2 of our framework is segmenting the overlapped nuclei region. To achieve this, we utilize the same SUNets as the ones used in the first stage. The difference is that we construct new generalized images as input images. These input images contain not only the original RGB images but also the binary masks predicted by Stage 1 as the fourth set of values. Due to the imbalanced distribution of overlapping nuclei regions and background regions, in the training step we employ focal loss (Lin et al., 2017), which is a method that was first proposed to address the sizable imbalance between the positive and negative distributions. Compared with the traditional cross entropy loss, focal loss introduces a function to measure the contribution of the hard and easily classified sample to the total loss. At the end of Stage 2, the segmentation result of overlapped regions is generated with pixel gray value of 0 (not overlapped regions) and 1 (overlapped region). Finally, nucleus instance segmentation results from the first stage are updated by adding the corresponding overlapped objects which have at least 10 overlapped pixels with the segmented objects derived from the second stage.

### Evaluation Metric

We evaluate our method by using two types of metrics: object-level and pixel-level metrics.

The commonly used Aggregated Jaccard Index (AJI) (Kumar et al., 2017) is utilized as object-level evaluation metric. The AJI is an extension of the Jaccard Index, and is defined as

$$\text{AJI}=\frac{\sum_{i=1}^{K}\left|{\text{GT}}_{i}⋂{\text{PRD}}_{j}^{*}\left(i\right)\right|}{\sum_{i=1}^{K}\left|{\text{GT}}_{i}⋂{\text{PRD}}_{j}^{*}\left(i\right)\right|+\sum_{l\in U}\left|{\text{PRD}}_{l}\right|}$$

where GT*_*i*_* (*i* = 1, 2,…, *K*) is a pixel belonging to ground truth (GT) nuclei object, PRD*_*j*_* (*j* = 1, 2,…, *L*) is a pixel belonging to the predicted nuclei objects. PRD*_*j*_*^∗^(*i*) is the connected component object from the predicted objects that have the maximum Jaccard Index with the GT nuclei, and *U* is the union of predicted objects that does not have intersections with any GT objects.

We also employ precision, recall rate, and F1-score (Sasaki, 2007) as the pixel-level evaluation metrics, which are defined as follows:

$$\text{precision}=\frac{\text{TP}}{\text{TP}+\text{FP}}$$

$$\text{recall}=\frac{\text{TP}}{\text{TP}+\text{FN}}$$

$$F1\_\text{score}=\frac{2\text{TP}}{2\text{TP}+\text{FN}+\text{FP}}$$

where FP, TP, and FN denote false positive, true positive, and false negative, respectively.

### Datasets

We evaluated our method by utilizing two publicly available datasets sourced from the Cancer Genome Atlas (TCGA)^1^ (Kumar et al., 2017) and the Triple-Negative Breast Cancer (TNBC) (Naylor et al., 2017). The two image datasets used herein are subsets of the TCGA and TNBC; the GT nuclear segmentation for these sets is manually performed by experts, and these datasets are widely used as the gold standard for nuclei segmentation studies.

The first dataset (selected from the TCGA database) (Tomczak et al., 2015) consisted of 30 hematoxylin and eosin (H&E) stained images. In this dataset, images were collected from seven different organs [bladder, breast, colon, kidney, liver, prostate, and stomach; we manually extracted 1,000 × 1,000-pixel size small patches from whole slide images (WSIs)] and used as the training (*n* = 16) and testing (*n* = 14) image sets.

The second dataset (selected from the TNBC database) contained 50 H&E stained images with 512 × 512 resolution. All images in the second dataset were extracted from 11 TNBC patients with multiple cell types including endothelial cells, inflammatory cells, and myoepithelial breast cells. We used this dataset to compare the performance of our nucleus instance segmentation with other popular methods.

### Network Implementation Details

All the experiments were conducted using TensorFlow (Abadi et al., 2016). Both SUNets used in the two-stage framework were trained using the same strategy–the learning rate used for training was 0.0001, stochastic gradient descent was used as an optimizer to minimize the loss function with momentum 0.9, and the batch size was set to 4. For the consideration of GPU memory limitation and performance, the input image size of all networks was set to 384 × 384 pixels. Additionally, we employed various augmentation techniques during the training process such as image rotation, vertical flipping, and horizontal flipping. Due to the sizable imbalance of overlapping and background regions, we trained the second Stacked U-Net with focal loss, whereas the first one was trained with cross-entropy loss. The networks were trained for 8 h on two NVIDIA Tesla P100 GPU cells for 20 epochs with CUDA 9.0 (NVIDIA, United States) library.

## Results and Discussion

**FIGURE 2:**
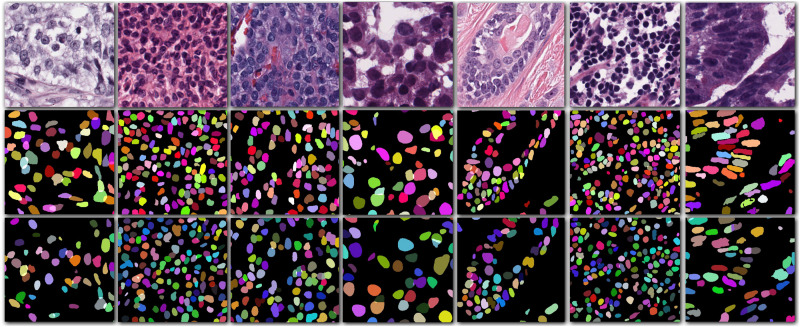
Cropped images from seven different organs (first row) with their corresponding ground truth (second row) and the segmentation result of our method (third row).

**FIGURE 3:**
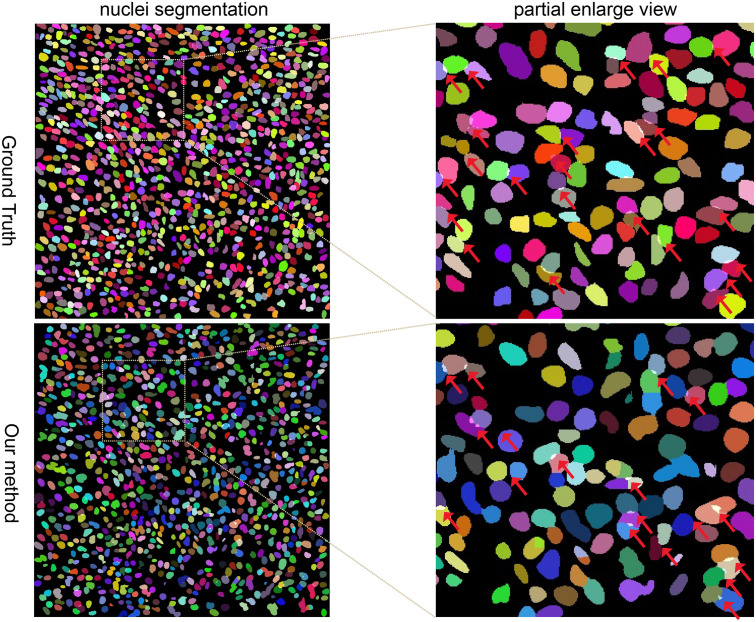
Randomly selected example of nuclei segmentation using our method. Each nucleus is randomly colored. First column: Segmentation of Ground Truth and our method. Second column: partially enlarged review of the nuclei segmentation. Red arrows point to the overlapped regions.

Our method outperformed present state-of-the-art methods on the two datasets (described in section “Datasets”) in the integrity of the segmentation of a single nucleus and the segmentation accuracy, and especially in the segmentation of overlapped nuclei regions. We compared our method against several deep learning based methods listed in Table 1, such as FCN-8 (Long et al., 2015), Mask R-CNN (He et al., 2015), U-Net (Ronneberger et al., 2015), CNN3 (Kumar et al., 2017), DIST (Naylor et al., 2019), SUNets, U-Net (DLA), a two-stage U-net (Mahbod et al., 2019), and two-stage learning U-Net (DLA) (Kang et al., 2019). In order to make the comparison objectively, we followed the same training and testing set split criteria suggested by Kumar et al. (2017). The results of the comparison confirmed the superiority of our method which achieved an average AJI of 59.65% and F1-score of 82.47% (Tables 1, 2 and Supplementary File S1). The performance results of the algorithms utilized in this comparison are sourced from the respective publication (Tables 1–3). Regarding the comparison of segmentation results (i.e., the extracted part of the whole image) of our method and the corresponding GT segmentation on different tissue types in the test image (displayed in Figure 2), our method was able to segment the majority of overlapped nuclei regions (the average AJI value of segmented overlapping regions was 0.3254) (Figure 3). The segmentation results of our algorithm are further illustrated in Figure 3, where we selected two examples of segmentation at random and compared them with the GT. We also applied our method on the TNBC dataset and compared the experimental results with other methods (Table 3)–DeconvNet (Noh et al., 2015), FCN-8 (Long et al., 2015), U-Net (Ronneberger et al., 2015), Ensemble method (Naylor et al., 2017), DIST (Naylor et al., 2019), and two-stage learning U-Net (DLA) method (Kang et al., 2019). Our method has the top AJI value (AJI = 0.621) but the second highest F1 score (F1 score = 0.806). This result demonstrates that our method has a high generalization ability since these images vary from tissue types to cell types. The values of AJI and F1 score of other methods in Tables 1–3 are taken from published works (Kang et al., 2019; Naylor et al., 2019).

**TABLE 1:** Comparison of AJI of different methods applied to the TCGA test set.

Organ	Bladder	Colorectal	Stomach	Breast	Kidney	Liver	Prostate	Overall
FCN-8 (Long et al., 2015)	0.5376	0.4018	0.5279	0.5598	0.5267	0.5045	0.5709	0.5171
Mask R-CNN (He et al., 2015)	0.5011	0.3814	0.6151	0.4913	0.5182	0.4622	0.5322	0.5002
U-Net (Ronneberger et al., 2015)	0.5403	0.4061	0.6529	0.4681	0.5426	0.4284	0.5888	0.5182
CNN3 (Kumar et al., 2017)	0.5217	0.5292	0.4458	0.5385	0.5732	0.5162	0.4338	0.5083
DIST (Naylor et al., 2019)	0.5971	0.4362	0.6479	0.5609	0.5534	0.4949	0.6284	0.5598
Stacked U-Net	0.6138	0.5188	0.5845	0.5605	0.5647	0.4594	0.5300	0.5474
U-Net (DLA)	0.6215	0.5322	0.5938	0.5747	0.5624	0.4642	0.5602	0.5584
A two-stage U-Net (Mahbod et al., 2019)	0.5706	0.4891	0.6545	0.5613	0.5755	0.4989	0.6316	0.5687
Two-stage learning U-Net (DLA) (Kang et al., 2019)	**0.6285**	0.5376	**0.6620**	**0.6096**	**0.6024**	0.5142	0.5720	0.5895
Ours	0.5926	**0.5586**	0.6541	0.5907	0.5926	**0.5346**	**0.6521**	0.**5965**

*The best results are shown in bold.*

**TABLE 2:** Comparison of F1 scores of different methods applied to the TCGA test set.

Organ	Bladder	Colorectal	Stomach	Breast	Kidney	Liver	Prostate	Overall
FCN-8 (Long et al., 2015)	0.8084	0.6934	0.7982	0.8113	0.5797	0.7589	0.8367	0.7552
Mask R-CNN (He et al., 2015)	0.7610	0.6820	0.8268	0.7481	0.7554	0.7157	0.7401	0.7470
U-Net (Ronneberger et al., 2015)	0.7953	0.7360	0.8638	0.7818	0.7913	0.6981	0.7904	0.7795
CNN3 (Kumar et al., 2017)	0.7808	0.7399	**0.8948**	0.7181	0.7222	0.6881	0.7922	0.7623
DIST (Naylor et al., 2019)	0.8196	0.7286	0.8534	0.8071	0.7706	0.7281	0.7967	0.7863
Stacked U-Net	0.8249	0.7685	0.8498	0.7990	0.7986	0.7276	0.7829	0.7930
U-Net (DLA)	0.8296	0.7756	0.8530	0.8025	0.7994	0.7296	0.7895	0.7970
A two-stage U-Net (Mahbod et al., 2019)	0.7599	0.7668	0.8912	0.8024	**0.8531**	**0.7938**	**0.8648**	0.8189
Two-stage learning U-Net (DLA) (Kang et al., 2019)	**0.8360**	0.7808	0.8629	**0.8183**	0.8022	0.7513	0.8037	0.8079
Ours	0.8217	**0.8135**	0.8690	0.8123	0.8251	0.7865	0.8451	**0.8247**

*The best results are shown in bold.*

**TABLE 3:** Quantitative comparison of different methods applied to the TNBC dataset.

Organ	Recall	Precision	F1-Score	AJI
DeconvNet (Noh et al., 2015)	0.773	**0.864**	0.805	–
FCN-8 (Long et al., 2015)	0.752	0.823	0.763	–
U-Net (Ronneberger et al., 2015)	0.800	0.820	0.810	0.578
Ensemble (Naylor et al., 2017)	**0.900**	0.741	0.802	–
Stacked U-Net	0.802	0.830	0.816	0.580
U-Net (DLA)	0.812	0.826	0.818	0.586
DIST (Naylor et al., 2019)	–	–	0.824	0.585
Two-stage learning U-Net (DLA) (Kang et al., 2019)	0.833	0.826	**0.829**	0.611
Ours	0.853	0.792	0.806	**0.621**

*The best results are shown in bold.*

## Conclusion

Nuclei segmentation has a wide utility in multiple biologically related tasks such as the quantitative analyses of the cellular constitution of tissues. Nuclei segmentation, however, is a notoriously challenging problem due to shape variation, imperfect slide digitalization, and the existence of overlapped or contact regions. In this study, we present a Stacked U-Net-based two-stage learning framework for nuclei segmentation of histopathological images. We divide the process of nuclei segmentation into the following steps: in the first step we segment the nuclei regions, and in the second step we divide the overlapping regions. Finally, nuclei instance segmentation results are updated by merging the two segmentation results. The results on two diverse public datasets show that our method outperforms most of the current standard segmentation methods and achieves state-of-the-art segmentation of not only the nuclei instances but also the overlapped regions.

## Data Availability Statement

Publicly available datasets were analyzed in this study. This data can be found here: https://nucleisegmentationbenchmark.weebly.com/ and https://zenodo.org/record/1175282/files/TNBC_NucleiSegmentation.zip.

## Author Contributions

YK and HL conceived the concept of the work. YK performed the data acquisition, the model design, and data analysis and drafted the manuscript. XW participated in model design. GG and XW contributed to the data interpretation and manuscript writing. HL and HZ supervised all aspects of the study. All authors read and approved the final manuscript.

## Conflict of Interest

The authors declare that the research was conducted in the absence of any commercial or financial relationships that could be construed as a potential conflict of interest.

## Footnotes

1 http://cancergenome.nih.gov/
